# Supplementary figures and images for: Investigating the immunomodulatory effects of honeybee venom peptide apamin in Drosophila platforms
Source: Infect Immun. 2025 Jun 5;93(7):e00131-25. doi: 10.1128/iai.00131-25 (PMC12234437; doi:10.1128/iai.00131-25)

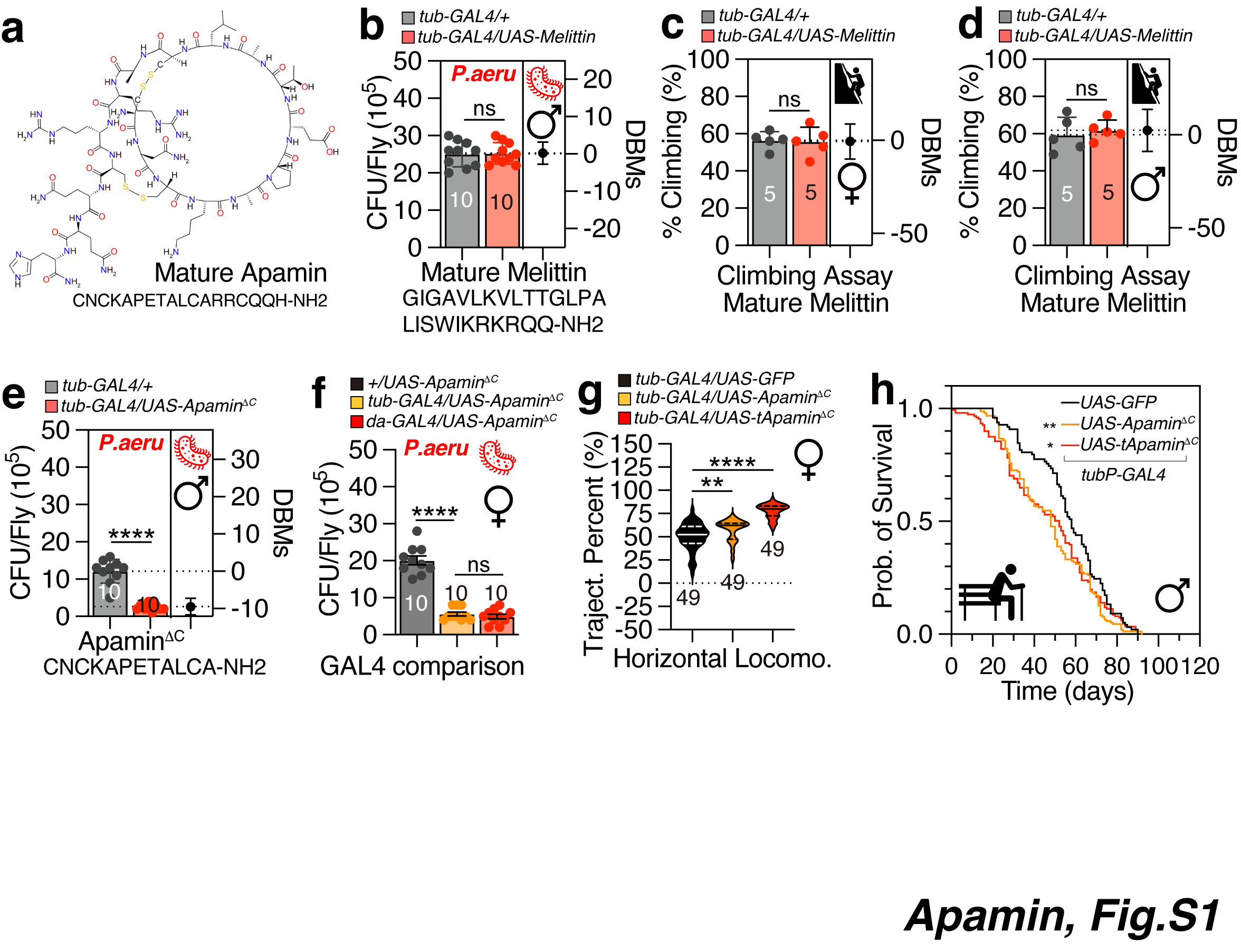

Supplement: Fig. S1 — Apamin structure, antimicrobial effects, fly survival, and locomotion. [file iai.00131-25-s0001.tif]

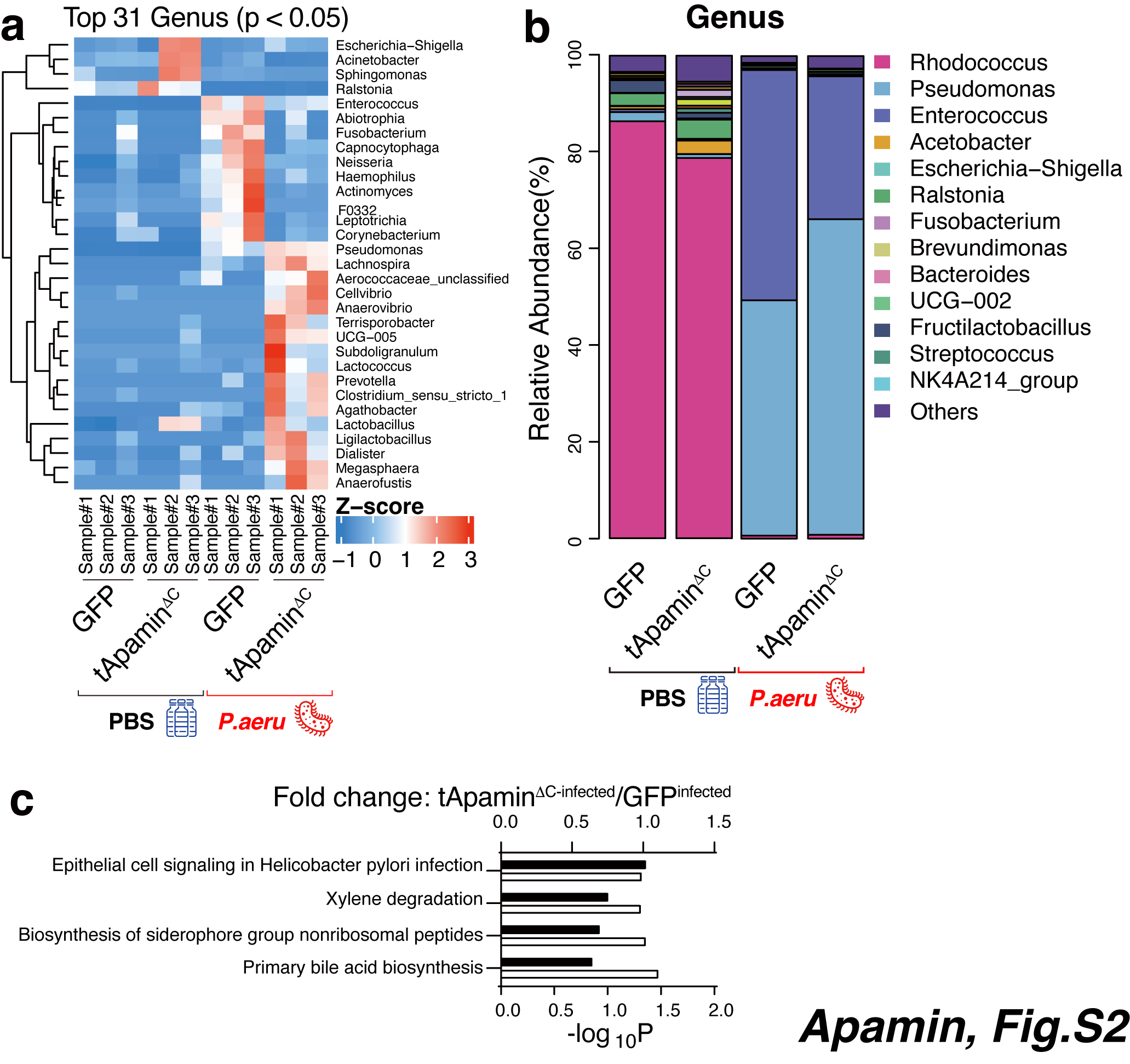

Supplement: Fig. S2 — Gut microbiome analysis (16S rRNA) of flies. [file iai.00131-25-s0002.tif]

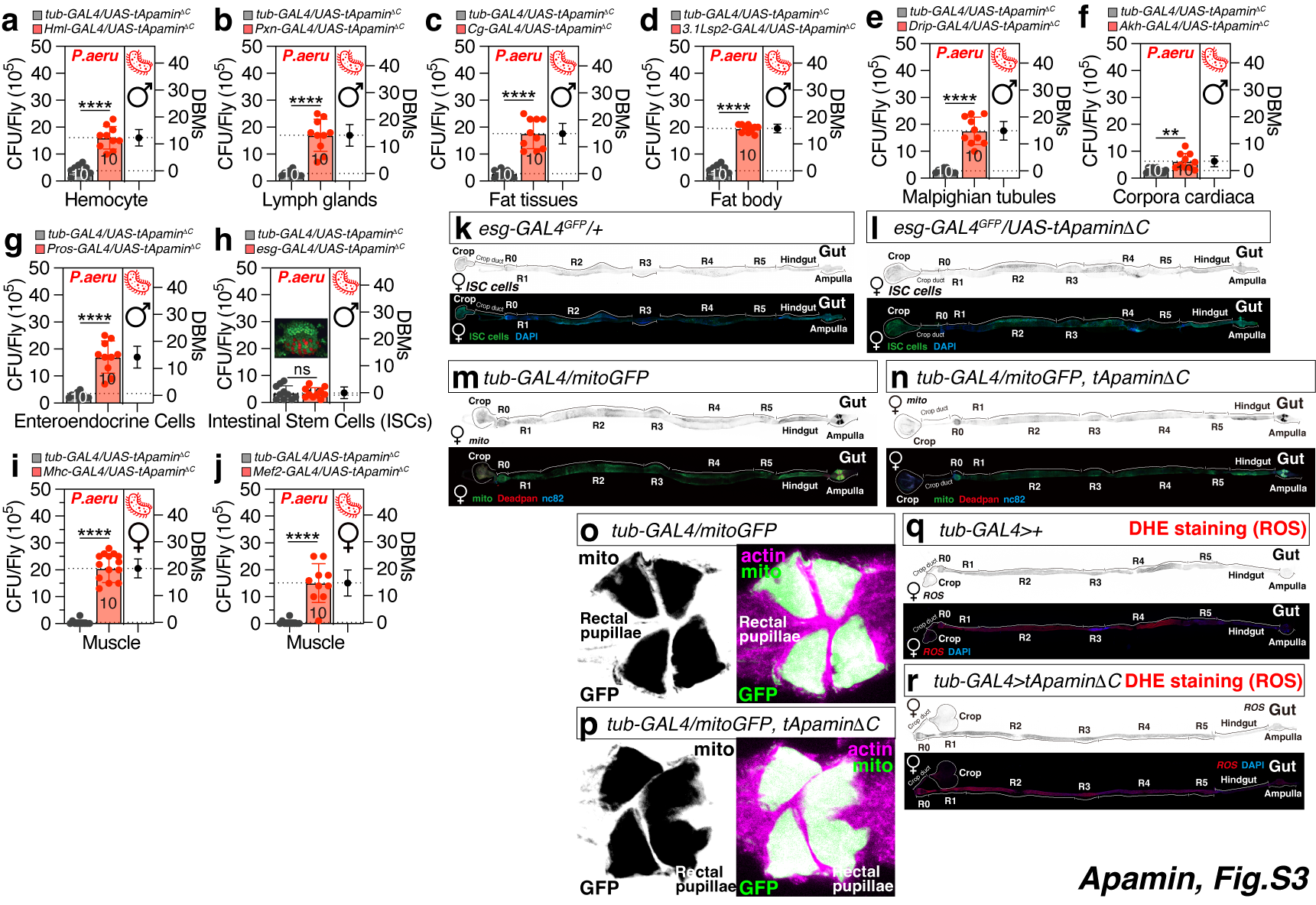

Supplement: Fig. S3 — Antimicrobial tissue screening and gut data in tApamin flies. [file iai.00131-25-s0003.tif]

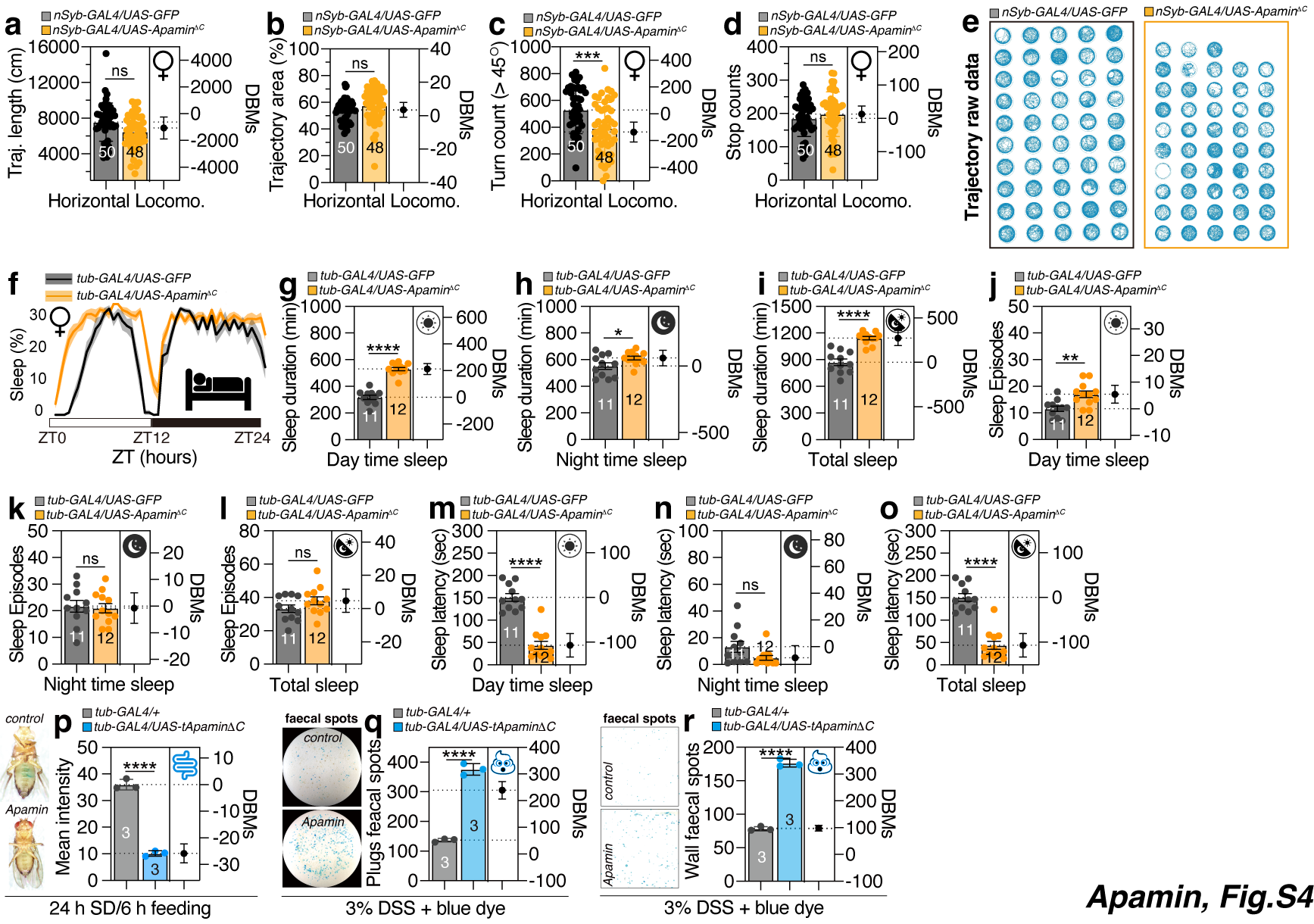

Supplement: Fig. S4 — Locomotion, sleep, and stress (Smurf) in Apamin flies. [file iai.00131-25-s0004.tif]

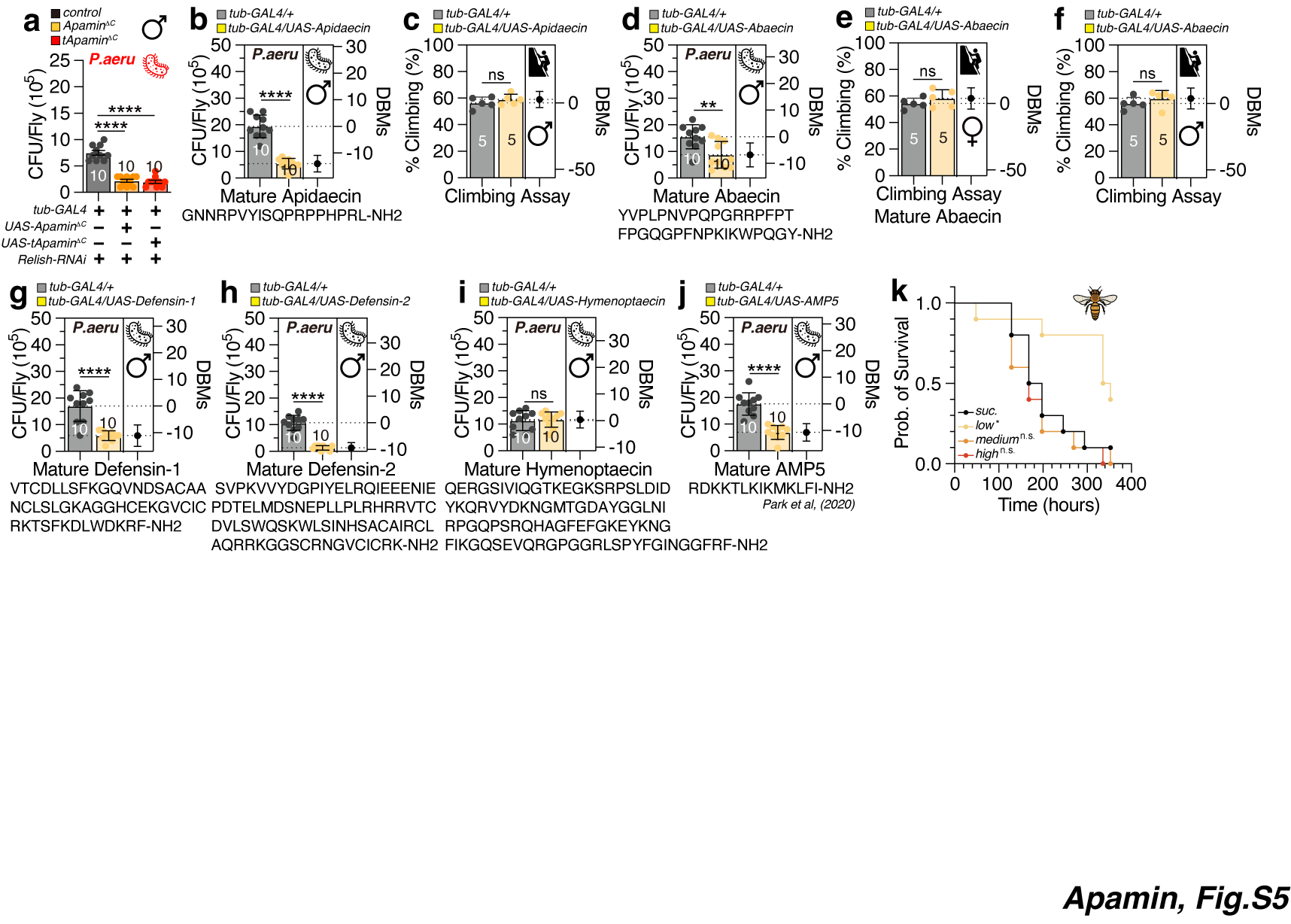

Supplement: Fig. S5 — Apamin effects on honeybee immunity and survival. [file iai.00131-25-s0005.tif]
