# Supplementary material for: Investigating the immunomodulatory effects of honeybee venom peptide apamin in Drosophila platforms
Source: Infect Immun. 2025 Jun 5;93(7):e00131-25. doi: 10.1128/iai.00131-25 (PMC12234437; doi:10.1128/iai.00131-25)
Supplement: Supplemental material — Supplemental figure and video captions. [file iai.00131-25-s0006.docx]

**Supplemental Figure titles and legends**

**Fig.S1: The chemical structure of apamin, the antimicrobial effect, survival curve and locomotion of flies expressing different honeybee VPs.**

**a,** Chemical structure of apamin and mature apamin amino acids sequence. **b,** Pathogen load of male flies expressing mature format melittin by *tub-GAL4* following a 12-hour oral feeding of *P. aeruginosa* culture. CFU stands for Colony-Forming Unit. DBMs represent difference between means, which is a statistical measure that quantifies the average discrepancy between two groups. (n=10) **c,** The climbing ability of female flies expressing mature format melittin by *tub-GAL4*. (n=5) **d,** The climbing ability of male flies expressing mature format melittin by *tub-GAL4*. (n=5) **e,** Pathogen load of male flies expressing Apamin^ΔC^ via *tub-GAL4*. (n=10) **f,** Pathogen load of female flies expressing Apamin^ΔC^ by *tub-GAL4*, da-GAL4 compared with control. (n=10) **g,** The trajectory percentage of female Apamin^ΔC^ and tApamin^ΔC^ flies compared with control flies. (n=49) **h,** The survival curve of control male flies and male flies expressing Apamin^ΔC^ and tApamin^ΔC^ via by *tub-GAL4* driver. (n=50)

**Fig.S2: 16S rRNA sequencing results of control flies and flies expressing tApamin^ΔC^.**

**a,** Heatmap of the top 31 significant genus (P value < 0.05, in the comparison of infected and uninfected, tApamin^ΔC^ versus control; n = 3 for each condition). **b,** Dendrogram and histogram of the bacterial composition at the genus level. **c,** Bacterial KEGG pathway prediction by PICRUSt2. Black bar: Foldchange of relative abundance; white bar: −log10P values for each KO term.

**Fig.S3: Tissue screening of antimicrobial effects in tApamin^ΔC^ expressing male flies and gut environment changes displayed.**

**a-h,** Pathogen load between male flies expressing tApamin^ΔC^ via **a,** *tub-GAL4* and *Hml-Gal4*, **b,** *Pxn-GAL4*, **c,** *Cg-GAL4*, **d,** *3.1Lsp2-GAL4*, **e,** *Drip-GAL4*, **f,** *Akh-GAL4*, **g,** *Pros-GAL4*, **h,** *esg-GAL4*, following a 12-hour oral feeding of *P. aeruginosa* culture. (n=10) **i,** Pathogen load between female flies expressing tApamin^ΔC^ via *tub-GAL4* and *Mhc-Gal4*. (n=10) **j,** Pathogen load between male flies expressing tApamin^ΔC^ via *tub-GAL4* and *Mef2-Gal4*. (n=10) **k, l,** The ISCs in full female fly gut between normal condition **(k)** and tApamin^ΔC^ expression flies **(l)** driven by *esg-GAL4* with *UAS-GFP*, fly guts were immunostained with anti-GFP (green), and DAPI (blue). **m-p,** The Dpn-positive neuroblast cells and mitochondria (*UAS-mitoGFP*) in full gut of female flies in normal **(m)** and tApamin^ΔC^ expressing conditions **(n)** by *tub-GAL4*, and the rectal pupillae part respectively **(o,p)**. Fly guts were immunostained with anti-Deadpan (red), anti-GFP (green), and DAPI (blue). **q,r,** DHE staining of ROS level in full female fly gut between control **(q)** and tApamin^ΔC^ expressing flies **(r)** driven by *tub-GAL4*. Fly guts were stained with DHE (red) and DAPI (blue) (repeated for 3 times).

**Fig.S4: Locomotion of neuronal Apamin^ΔC^ expressing fly, sleep pattern of pan-expressed Apamin^ΔC^ fly and Smurf related results induced by stress.**

**a,** The trajectory length, **b,** trajectory area, **c,** rotation times, **d,** stop counts, and **e,** trajectory diagram of female flies expressing Apamin^ΔC^ by *nSyb-GAL4* (n=48) compared with controls (n=50). **f-o,** Sleep profiles (average proportion of time spent sleeping in consecutive 30-min segments during a 24-h LD cycle) and quantification of female flies expressing Apamin^ΔC^ expression by *tub-GAL4* driver (n=11 for controls, n=12 for Apamin^ΔC^ expressing flies) **(f)**. Quantification of sleep durations for Apamin^ΔC^ expression flies **(g-i)**. Quantification of sleep episodes for Apamin^ΔC^ expression flies **(j-l)**. Quantification of sleep latency for Apamin^ΔC^ expressing flies **(m-o)**. **p,** The mean intensity of Smurf assay flies expressing tApamin^ΔC^ compared with controls after 24 hours sleep deprivation and 6 hours dye feeding. The experiment was repeated three times and one representative figure was shown of each condition. **q,** The counts of facets on plugs containing flies treated with a 3% DSS feeding with blue dye. The experiment was repeated three times and one representative figure was shown of each condition. **r,** The counts of facets on tube wall of flies treated with a 3% DSS feeding with blue dye. The experiment was repeated three times and one representative figure was shown of each condition (repeated for 3 times).

**Fig.S5: Possible immune genes and AMPs related, and survival of honeybees applied to apamin in different concentrations.**

**a,** Pathogen load of male flies expressing Apamin^ΔC^ and tApamin^ΔC^ by *tub-GAL4*, with a knockdown of *Relish* compared with control. (n=10) **b,** Pathogen load (n=10) and **c,** Climbing ability (n=5) of controls and male flies expressing Apidaecin by *tub-GAL4* following a 12-hour oral feeding of *P. aeruginosa* culture. **d,** Pathogen load of controls and male flies expressing Abaecin by *tub-GAL4*. (n=10) **e,** Climbing ability of controls and female flies expressing Abaecin via *tub-GAL4*. (n=5) **f,** Climbing ability of controls and male flies expressing Abaecin via *tub-GAL4*. (n=5) **g-j,** Pathogen load of controls and male flies expressing Defendin-1 **(g)**, Defensin-2 **(h)**, Hymenoptaecin **(i)**, and AMP5 **(j)** by *tub-GAL4* following a 12-hour oral feeding of *P. aeruginosa* culture. (n=10) k, The survival curve of honeybees infected orally by *P. aeruginosa*, following sucrose administration as control and three different concentrations of apamin administration. Control (black) (n=9) compared with low concentration apamin (yellow) (n=9), medium concentration apamin (orange) (n=10), and high concentration apamin (red) (n=10) administration.

**Movie.S1: Honeybees after apamin administration.**

**Movie.S2: Honeybee apamin and bacteria administration.**
